# Acceptability of a complex team-based quality improvement intervention for transient ischemic attack: a mixed-methods study

**DOI:** 10.1186/s12913-021-06318-2

**Published:** 2021-05-12

**Authors:** Teresa M. Damush, Lauren S. Penney, Edward J. Miech, Nicholas A. Rattray, Sean A. Baird, Ariel J. Cheatham, Charles Austin, Ali Sexson, Laura J. Myers, Dawn M. Bravata

**Affiliations:** 1 Department of Veterans Affairs (VA) Health Services Research and Development (HSR&D) Precision Monitoring to Transform Care (PRIS-M) Quality Enhancement Research Initiative (QUERI), Richard L. Roudebush VA Medical Center, Indianapolis, IN USA; 2 VA HSR&D Center for Health Information and Communication (CHIC), Richard L. Roudebush VA Medical Center, Indianapolis, IN USA; 3 Department of General Internal Medicine, Indiana University School of Medicine, Indianapolis, IN USA; 4 Regenstrief Institute, Indianapolis, IN USA; 5 Elizabeth Dole Center of Excellence for Veteran and Caregiver Research, San Antonio, TX USA; 6 Department of Medicine, University of Texas Health San Antonio, San Antonio, TX USA; 7 Department of Neurology, Indiana University School of Medicine, Indianapolis, IN USA

**Keywords:** Acceptability, Complex intervention, Quality improvement, Theoretical framework of acceptability, Temporality

## Abstract

**Background:**

The Protocol-guided Rapid Evaluation of Veterans Experiencing New Transient Neurologic Symptoms (PREVENT) program was a complex quality improvement (QI) intervention targeting transient ischemic attack (TIA) evidence-based care. The aim of this study was to evaluate program acceptability among the QI teams and factors associated with degrees of acceptability.

**Methods:**

QI teams from six Veterans Administration facilities participated in active implementation for a one-year period. We employed a mixed methods study to evaluate program acceptability. Multiple data sources were collected over implementation phases and triangulated for this evaluation. First, we conducted 30 onsite, semi-structured interviews during active implementation with 35 participants at 6 months; 27 interviews with 28 participants at 12 months; and 19 participants during program sustainment. Second, we conducted debriefing meetings after onsite visits and monthly virtual collaborative calls. All interviews and debriefings were audiotaped, transcribed, and de-identified. De-identified files were qualitatively coded and analyzed for common themes and acceptability patterns. We conducted mixed-methods matrix analyses comparing acceptability by satisfaction ratings and by the Theoretical Framework of Acceptability (TFA).

**Results:**

Overall, the QI teams reported the PREVENT program was acceptable. The clinical champions reported high acceptability of the PREVENT program. At pre-implementation phase, reviewing quality data, team brainstorming solutions and development of action plans were rated as most useful during the team kickoff meetings. Program acceptability perceptions varied over time across active implementation and after teams accomplished actions plans and moved into sustainment. We observed team acceptability growth over a year of active implementation in concert with the QI team’s self-efficacy to improve quality of care. Guided by the TFA, the QI teams’ acceptability was represented by the respective seven components of the multifaceted acceptability construct.

**Conclusions:**

Program acceptability varied by time, by champion role on QI team, by team self-efficacy, and by perceived effectiveness to improve quality of care aligned with the TFA. A complex quality improvement program that fostered flexibility in local adaptation and supported users with access to data, resources, and implementation strategies was deemed acceptable and appropriate by front-line clinicians implementing practice changes in a large, national healthcare organization.

**Trial registration:**

clinicaltrials.gov: NCT02769338.

**Supplementary Information:**

The online version contains supplementary material available at 10.1186/s12913-021-06318-2.

## Background

Program acceptability by front-line users of an evidence-based, quality improvement (QI) program is a hallmark of successful implementation [1]. Indeed, in the United Kingdom (UK), the Medical Research Council (MRC) has included program acceptability in its published guidance for appropriate methods for designing and evaluating complex interventions. More recently, program acceptability has been conceptualized as a program that meets the approval of the users, is appealing and likeable and its use is welcomed [2, 3]. More than liking a program, a systematic review of acceptability in 43 healthcare interventions concluded that “acceptability is a multi-faceted construct that reflects the extent to which people delivering or receiving a healthcare intervention consider it to be appropriate, based on anticipated or experienced cognitive and emotional responses to the intervention.” [4] Thus, the theoretical framework of acceptability (TFA) was developed as a multi-faceted construct, consisting of seven domains: (1) affective attitude, feelings about the intervention, (2). burden, perceived effort required to participate in the intervention, (3). perceived effectiveness, perceived likelihood to achieve the intervention’s purpose, (4). Ethicality, good fit with an individual’s value system, (5). intervention coherence, extent to which the participant understands the intervention and how it works, opportunity costs, extent to which the participant understands the intervention and how it works, and self-efficacy, participant’s confidence that she can perform the behavior(s) required to participate in the intervention [4].

Designing and implementing an acceptable program among targeted stakeholders is often a key aim of a QI program to promote program local adaptation and program uptake. However, less is known on how to design for program acceptability for complex interventions involving multiple components among multiple clinical users in a real world, healthcare system. Therefore, we applied the TFA to guide a program evaluation of its acceptability by its users, the QI teams, based on their experience with the Protocol-guided Rapid Evaluation of Veterans Experiencing New Transient Neurologic Symptoms (PREVENT) program.

### Objectives

The PREVENT program was designed to address systemic barriers to providing timely guideline-concordant care for patients with transient ischemic attack (TIA) including: lack of protocols, clinical uncertainty about TIA care, and absence of performance data [5–7]. The specific aim of this current project was twofold: 1) to evaluate the PREVENT program QI teams’ acceptability of the intervention across implementation phases over time, and 2). to examine factors associated with the multiple facets of acceptability.

## Methods

### Design and setting

The clinical trial design and protocol have been previously reported [8]. PREVENT was a stepped-wedge implementation trial with six geographically diverse sites within the U.S. Veterans Health Administration (VA). PREVENT’s sites were pragmatically allocated to 12-month active implementation in three waves, with two facilities per wave followed by a sustainment period. Both the trial and the evaluation were reviewed and approved by the Indiana University local institutional review (IRB) board #1511914238 and the VA research and development committee. The IRB approved both a waiver of written informed consent and the obtainment of verbal consent for clinical provider interviews. No changes to the trial design were made after trial commencement. Facilities were not randomized.

### Quality improvement intervention

The rationale and methods used for the development of the PREVENT intervention have been described elsewhere [8]. The clinical provider-facing, QI intervention in PREVENT was based on a prior systematic assessment of TIA care performance at VA facilities nationwide as well as an evaluation of barriers and facilitators of TIA care performance using four sources of information: baseline quality of care data [5], staff interviews [6], existing literature [9–13], and validated electronic quality measures [5]. The PREVENT QI intervention included five components: quality of care reporting system, clinical programs, professional education, electronic health record tools, and QI support including a virtual collaborative.

### Implementation strategies

PREVENT employed a bundle of three primary implementation strategies which were previously reported: [14, 15] (1) team activation via audit and feedback [16], reflecting & evaluating, planning, and goal setting [17]; (2) external facilitation (EF) [16, 18]; and (3) building a community of practice (CoP) [19, 20]. In addition, PREVENT allowed for local adaptation of its intervention components with a high degree of flexibility.

Active implementation of PREVENT at each site began with a full-day kickoff meeting facilitated by the coordinating site and included multidisciplinary staff members from the participating site involved in TIA clinical care. Using approaches from systems redesign [21, 22], site team members brainstormed about barriers to providing highest quality of care, identified solutions to address barriers, ranked solutions on an impact-effort matrix, and developed a site-specific action plan that included high-impact/low-effort activities in the short-term plan and high-impact/high-effort activities in the long-term plan. Local QI plans were entered into the PREVENT Hub (a centralized virtual platform which included an audit and feedback mechanism and quality improvement resources) [15] metrics were tracked on the Hub allowing teams to monitor performance over time.

During the one-year active implementation period, the site team members joined monthly PREVENT virtual collaborative conference calls which served as a forum for sites to share progress on action plans, articulate goals for the next month, and review new evidence or tools. The sites’ teams had access to the data Hub, an interoperative web based platform used to provide feedback on quality performance,and the collaborative calls during sustainment after active implementation; however, during sustainment they received no implementation assistance from the external facilitator.

### Participants

We began with a purposive sample at the VA medical facilities which snowballed based on clinical team participation at each of the six facilities. Clinical providers involved in the provision of TIA care at the six PREVENT sites volunteered to participate in the local PREVENT Quality Improvement program. All six clinical champions provided the names of their respective team members. The facility and participant flow has been previously reported [23]. Participants were approached by email and telephone. There were no presence of non-participants.

### Outcomes

We employed a mixed-methods design to measure and evaluate program acceptability based on the of its users, the QI teams. Multiple data sources were triangulated for this evaluation using the Theoretical Framework of Acceptability (TFA) [4]. We conducted 29 onsite, semi-structured interviews during active implementation with 32 participants at 6 months and 30 participants at 12 months (see Table 1).

During the six-month interviews (see Additional File 1), we queried participants on the acceptability and perceptions of each of the five PREVENT components: (e.g., “*How helpful was it, if at all, for you to have direct access to the PREVENT facilitator?” “How were you able to use any of the existing PREVENT materials provided by the national program? Which ones?” “How did you adapt to your local facility?”).* Moreover, we queried on the multiple components of the TFA. We asked about the following:the burden; the affective attitudes about the PREVENT program; the in-kind,opportunity costs associated with local PREVENT implementation; the QI teams’ experiences and coherence with PREVENT and the implementation strategies; and the appropriateness,ethics & values- of the evidence-based program. We also queried participants within the six teams on their progress in implementing their action plans during active implementation.

At 12 months (see Additional File 2), we queried about the PREVENT program perspective. For example, *“In terms of the implementation activities you have undertaken, which have been most impactful on local TIA care?” “Looking back, what would you have done differently if you knew then what you know now about PREVENT implementation?” “Thinking about future PREVENT implementation, what advice would you give to personnel at a VAMC who wants to implement PREVENT?”*

The evaluation team which excluded the external facilitator followed-up with 19 participants, including site clinical champions and at least one or more team member, during program sustainment (see Additional File 3) with similar questions from the 12-month interviews. In addition, we reviewed each team’s quality data and asked about their perceived effectiveness and sustainment. We also conducted debriefing meetings among members of the national coordinating center (facilitators and evaluators) after onsite visits and monthly virtual collaborative calls with representatives from the 6 teams. The duration of each interview ranged between 30 and 45 min.

Interim analyses and stopping guidelines: N/A

Outcomes and estimation: N/A

Binary Outcomes: N/A

Harms: No harms were reported.

All interviews and debriefings were audiotaped and transcribed. All interviews were de-identified and imported into NVivo12 software for data coding and analyses [24]. De-identified files were qualitatively coded and analyzed for common themes and acceptability patterns. In addition, a team of TD, LP, AC, and SB further reviewed and evaluated key codes affiliated with acceptability and appropriateness of the PREVENT program across QI teams and across time. Debriefings were reviewed for supporting evidence of themes. Overall program satisfaction ratings were assessed during the 12 month interviews using a 1–7 response set where a score of “7” indicated highly satisfied. *“Overall, on a 1-7 scale where 1 indicates “not at all” and a 7 indicates “highly satisfied”, how satisfied were you with the PREVENT program?” “Please explain why you chose your rating?”*

To develop the qualitative database, we followed team procedures as follows [25]. Each transcript was independently coded by at least two project team members using a common codebook that included selected items related to TFA acceptability (e.g.,” acceptability;” “appropriateness;” “barriers;” “self-efficacy”). Coders reconciled coding through discussion until a common understanding was reached; unresolved questions and codebook refinement were discussed during weekly project meetings. Reconciled coded files were merged into a final dataset for analysis.

### Analysis plan

We described participant endorsements of the kickoff planning meeting elements. We identified emergent themes and patterns and illustrated with direct quotations. We conducted case comparison analyses and compared codes across sites and by disciplines (e.g., neurology, pharmacy). We conducted mixed-methods, matrix analyses. We compared program satisfaction ratings at the end of active implementation by roles on QI teams (champions vs team support) and subsequently we compared acceptability and appropriateness related codes by satisfaction ratings to elucidate upon program and contextual related factors associated with perceived program acceptability. We further evaluated the temporality effect of acceptability from active implementation through program sustainment. Finally, we assessed perceived effectiveness on the local programs by evaluating which factors made the greatest impact.

## Results

### Participants - quality improvement [QI] team and facility Characteristics

In Table 1, we presented the total number of interviews completed by facility and by participants’ role at 6 and 12 months of active implementation. We completed 30 interviews with 35 participants at 6 months and 27 interviews with 28 key informants at 12 months. The QI teams represented the regions of the national VHA system. Two-thirds of the QI teams were led by stroke neurologists or general neurologists and the remaining one-third of sites were led by emergency medicine nursing or a systems redesign specialist.

**Table 1:** The PREVENT Quality Improvement Interviewees and Facility Characteristics

INTERVIEW	SITES BY WAVES													
CHARACTERISTICS	INTERVIEW PERIODS													
	Wave 1				Wave 2				Wave 3				Overall	
	101		102		103		104		105		106		All	All
	6	12	6	12	6	12	6	12	6	12	6	12	6	12
Total Number of Interviews by Time Period	6	5	4	3	7	4	4	5	6	5	3	5	30	27
Total Number of Interviewees by Time Period	7	5	5	3	8	4	6	6	6	5	3	5	35	28
Emergency Medicine physicians	1	1	1	1	1	1	1	2	1		1	1	6	6
Neurologists	1	1	2	1	1	1	1	1	1	1	1	1	7	6
Nurses (ED, Education, Telehealth)	3	2	0	0	2	1	0	0	1	1	0	1	6	5
Pharmacists	2	1	1	1	1	0	3	2	1	2	0	0	8	6
Clinical Informaticians/ System Redesign	0	0	1	0	1	0	0	0	1	1	1	1	4	2
Hospitalists	0	0	0	0	1	1	0	0	1	0	0	1	2	2
Radiologists	0	0	0	0	2	0	1	1	0	0	0	0	3	1
Years at VA Mean (SD) [Range] (m = months) (y = years)	4.07 (2.90) 3 m-9y	4.45 (2.82) 9 m-9y	13.69 (10.69) 9 m-30y	15.25 (11.94) 9 m-30y	11.44 (6.41) 2.5-21y	11.25 (6.83) 3-22y	4.58 (3.91) 1 m-10y	11.62 (12.61) 1 m-36y	5.75 (4.06) 1.5-14y	6.5 (4.39) 3-14y	6.5 (3.5) 3y-10y	7.33 (3.09) 3-10y	7.66 (6.78) 1 m-30y	9.13 (8.81) 1 m-36y
Facility Region	South		Northeast		Southeast		Midwest		West		Southeast		VHA	
Implementation Lead Service	Stroke Neurologist		Neurologist		Emergency Medicine RN		Stroke Neurologist		Stroke Neurologist		Systems Redesign & Stroke Neurologist		VHA	
Implementation Lead Satisfaction	7		7		7		7		6.5		7		6.93 (0.17) [6.5–7]	
Mean (SD) [Range] Overall Team Program Satisfaction (12 months only)	6.6 (0.49) [6, 7]		6.33 (0.47) [6, 7]		6.17 (0.85) [5–7]		5.67 (0.98) [4–7]		5.9 (0.49) [5–7]		6.83 (0.24) [6.5–7]		6.23 (0.75) [4–7]	
Baseline Without-Fail Rate (TIA performance)	55.2%		38.5%		16.3%		50.0%		33.3%		38.7%		–	

Note. Participants were identified as clinical team members who participated in PREVENT program during the active implementation period as noted by the site clinical champion and completed a 6-and/or 12-month interview. Among the 41 unique participants, 3 were missing “years worked at the VA.” Overall Program Satisfaction was assessed with a single item, “Overall, how satisfied are you with the PREVENT program?” Response set ranged from 1 to 7 where 7 denotes “extremely satisfied”

Years at VA refers to the number of years participants have practiced at their VA site. The mean, standard deviation, and range are provided for each site in addition to the total mean, standard deviation, and range for all participants. The N value is also provided for “Years at VA.” This value includes all participants who provided an answer for “years at VA” in their interview. Participants who did not provide an answer were counted as missing. Total number of missing “Years at VA for 6 months” = 3 and total number missing “Years at VA for 12 months” = 4

Satisfaction Score refers to the score provided by participants during the 12-month interview (scale 1–7). The mean, standard deviation, and range are provided for each site in addition to the total mean, standard deviation, and range for all participants. The N value is also provided for “Satisfaction Score.” This value includes all participants who provided a satisfaction score during his/her 12-month interview. Total number who were not asked Satisfaction Score and marked as missing = 4

### Active implementation

#### Team PREVENT overall satisfaction ratings at 12 months after active implementation

Team mean satisfaction rating is reported in Table 1. Overall Program Satisfaction was assessed with a single item, “Overall, how satisfied are you with the PREVENT program?” Response set ranged from 1 to 7 where 7 denotes “extremely satisfied.” The mean QI team satisfaction rating ranged from 5.67 to 6.83 indicating high overall team satisfaction with the PREVENT program. The level of reported satisfaction differed by site champion compared to rest of the local QI team. Across the 6 QI teams, 5/6 [83.3%] site champions reported a score of [7] “extremely satisfied” with the PREVENT program (see Table 1) Moreover, team PREVENT satisfaction did not vary by baseline quality performance. All teams found the PREVENT program acceptable.

The following quotations illustrated the differential in ratings between the team champion and team members:

*“I was highly satisfied, a 7, because I do think that it [PREVENT] motivated us to look at what was going on and to try to make some positive changes and to work as a team.”* [102 12m 3 SV-Site Champion].

*“I was probably less encouraging about starting than our stroke director [site champion] … because I just wasn’t sure what we were going to get out of it. Although I think I was wrong. I think that we actually did get a lot [out] of it [PREVENT], and it was worth the effort that we did put into it.”* [102 6 m 1 SV-Team Member].

Some reported the program was acceptable because a local, collaborative, multidisciplinary team emerged where previously one clinician had been responsible for improving cerebrovascular care at a facility.

*“ … we’ve been actually very happy with all this data coming in and putting it together because it’s been very good for us tracking as well as very good for us as a department to show the hospital that initiative like this [PREVENT] count. … the combination of getting different services together, having different goals so that everybody is able to kind of collaboratively accomplish, tracking the data and the web site [data Hub], and having the education from the calls. So all four of those have been fantastic.”* [104 12 m 3 SV].

Other teams reported that they were appreciative of the QI support and the opportunity to learn from other QI teams in the VHA system*. “I thought that [PREVENT] was a good program. I enjoyed working with it. I had time constraints, but I think that it was great to hear from other programs and what they’re doing, and I’ve interacted individually…*” [102 12 m 3 SV].

#### Team PREVENT acceptability guided by the theoretical framework for acceptability (TPA)

**Fig. 1:**
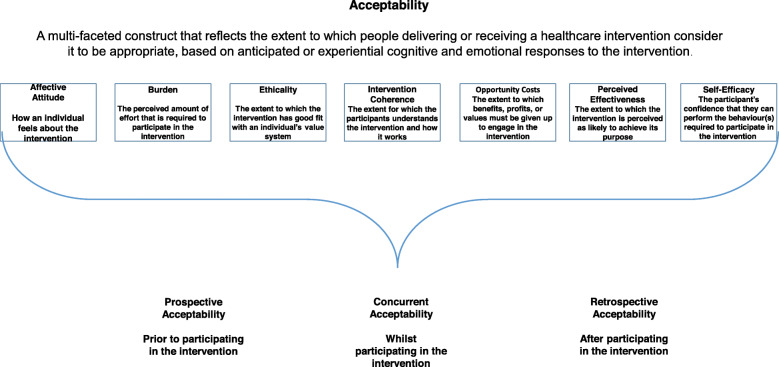
The Theoretical Framework of Acceptability. The Theoretical Framework of Acceptability is comprised of seven component constructs based on a systematic literature review [4]. The constructs are presented alphabetically with definitions. Constructs are considered concepts and components are considered aspects of the constructs. Figure 1 was originally published in open access from Sekhon et al. [4] BMC HSR publication (see References)

Overall, all six QI teams perceived the PREVENT program as acceptable during active implementation. Given that acceptability has been shown to be multifaceted [4], we applied each of the seven components of the multifaceted TPA (see Fig. 1) to the PREVENT program acceptability and presented each component with supportive data from the six QI teams to illustrate each component.

#### TPA affective attitudes

Denoted as how a participant “feels” about a program [4], the PREVENT program was well liked and appealing, deemed a very positive experience, and viewed as a professional quality program.

*“I have really enjoyed them [collaborative calls] … You could learn all kinds of things that might not even pertain to [my service area] but might be helpful to me to provide care. I thought that [the data Hub] was very organized to where if I needed to get something quickly and refer to it, it was very organized to where I could get to that quickly, and it wasn’t something that was cumbersome … [In the library on the Hub] I referred to those [guidelines] a couple of times just because I knew that it was an easy spot that I could get* [101 12 m 3 SV].

“*I was highly satisfied. This has been a great experience. It wasn’t without its frustrations, but those frustrations were not from PREVENT. Those frustrations were just the facility side here and just trying to make people see what areas that they’re accountable for …* [103 12 m 2 SV].

#### TPA burden

The perceived amount of effort that is needed to participate in the program was considered a burden [4]. During pre-implementation, leadership at several facilities were concerned about the potential burden of PREVENT on their clinical staff. In response the clinical champion would present the argument that PREVENT would facilitate their quality of care and that it was an opportunity worth the cost to engage in the program. Across active implementation, the QI Teams had expressed at times their participation burden.

“ … *it was with the process and the internal factors that we had involved with getting someone to be a consistent champion for Pharmacy. And so I think those very initial struggles kind of still weigh on my mind, and I’m wondering how could we have prevented that*.” [105 12m 5 SV]

Team members with leadership positions within the organization pointed out the need to justify time spent on PREVENT. *“The expectation to get on the Hub and just stay …*. *Let’s all stay caught up with the Hub - It’s not realistic.”* [103 12 m 1 SV] These leaders; however often supported their QI teams spending time on PREVENT elements.

At times during active implementation the burden became a source of frustration as the team efforts placed in making practice changes did not translate into immediate changes on the performance outcomes shown in the data Hub and this became a source of frustration among team participants. “*Well, I think that I got really frustrated with the without-fail rate because … I just felt that I was working so hard, and I was like trying to cover all of these holes … Our without fail rate was like zero for the month, … I really just wanted to quit [PREVENT] because I just was really frustrated* …. T*he external facilitator RN was really helpful and encouraging* …. [by giving suggestions on possible interpretations of the performance data]. … *then the next month was better*. [103 12 m 2 SV].

For some, the effort required of participation was realized by team participants soon after the kickoff. Teams which experienced a slower start during active implementation reported that PREVENT implementation was difficult.

“*I think it [kickoff] was a little hard … Because we didn’t have a full team, it just took a long time to get from there to actually doing anything because I was trying to field the team and get them to commit the time. There was a lot of work for two to three months of getting all of the players that we thought should be there to meet with you guys, and then only four of us could come [to the kickoff].*” [105 6 m 6 SV].

Despite the level of burden reported, over time in active implementation teams reorganized, emerged and participated in implementation activities during the last half of active implementation between 6 and 12 months and accomplished most of their action plans. Thus, after these accomplishments, the team acceptability ratings were more positive at 12 months compared to at 6 months.

“ … *even when we you know would have an idea, figuring out who we needed to talk to was often a very complicated and lengthy process. … So I feel like we were really slow to ramp up.* [105 12m 12 SV].

#### TPA ethicality

The degree to which the intervention has good fit with an individual’s values [4], participants perceived PREVENT as a good fit with their values and that their patients deserved this timely, evidence-based program.

*“So I think that the (PREVENT) experience itself was extremely valuable, and I think that the opportunity was something that there wasn't something else like it in the VA, and so I appreciated …. So I think that from a facility standpoint, we appreciated shining a light on something and giving us like tools and the opportunity to like self-reflect on how to change something that’s very important …”* [106 SUS 1 SV]

“*I have really enjoyed them [collaborative calls] … You could learn all kinds of things that might not even pertain to [my service area] but might be helpful to me to provide care...I think that we all learned a lot. [PREVENT] improved patient care, which was obviously most important I would say … without any downside to it.”* [101 12 m 3 SV].

#### TPA intervention coherence

This construct refers to the degree in which the participant comprehends the program and how it works [4]. Team members with previous QI experience and those who used data to improve the quality of care realized early in active implementation how useful the PREVENT QI program was with its ready to use resources, tools, and access to available site level quality data. Pharmacists were such team members and discussed after 6 months of active implementation how easy it was for them to use the PREVENT data Hub and how the PREVENT data metrics were aligned with their scope of practice.

“*Coming from research, education and my [pharmacy] clinical arena with my training and background from another VA, I was really happy that we were doing this [PREVENT] here because it kind of aligns with what the kind of environment I’m used to which is quite progressive and data-driven and … metrics and development using technology and dashboards … so I was very enthusiastic and I’m still enthusiastic.* [103 6 m 6 SV].

Pharmacists were members of all six QI teams and reported familiarity with data and ease of use of the PREVENT data Hub across the teams.

“*I thought that [the data Hub] was very organized to where if I needed to get something quickly and refer to it, it was very organized to where I could get to that quickly, and it wasn’t something that was cumbersome.”* [101 12 m 3 SV].

We observed that most of the site champions and their colleagues understood the targeted evidence-based practices for acute TIA care at pre-implementation. The challenge at times for some was to get buy in by their colleagues to place efforts on improving the quality of care.

*“I think the biggest thing is you’ve got to get buy in and get people who are going to be honestly motivated to go through it, and not just necessarily just pick one of the names [of staff] out of a hat. But you really need to have a multidisciplinary group [to represent all the services involved in the EBPs] who’s going to put their honest efforts into it.”* [102 SUS 1 SV].

“… *then to actually get the buy-in of the neurologists, that’s really where the shift came. Once we got the neurologists to buy into it [neurology based EBP], that’s when the shift happened [in quality improvement]. And so getting them [physicians] on board, on agreeing to it [PREVENT], definitely facilitates it [PREVENT]. Otherwise it’s just a push kind of thing. It’s people individually pulling*.” [103 12 m 4 SV].

#### TPA opportunity costs

Defined as the “extent to which benefits, profits, or values must be given up to engage in the intervention.” [4] The local adaptation and uptake of PREVENT utilized multiple FTE across time with repeating efforts to locally market the program. In one team they made the decision to utilize two different team members to educate and spread the PREVENT program within a facility to uptake the PREVENT program.

*“I would say there can never be enough education for your providers and your pharmacists. And repeat messaging is really important. What we found is that the ground rounds implementation [by clinical champion] was great, but it kind of fell off people’s radar like 2 months later. So I had our pharmacy resident go back and remind people again and so I think that is really important especially for sites like us who don’t have a whole lot of TIA patients anyway.”* [105 12 m 5 SV].

In another team a service chief simply told the clinical front-line to change a specific practice to improve the quality. *“I told people we had to do that [PREVENT] because we were told to … Have a good group of people who-they understand sometimes we have to do things that we don’t want to always do.”* [104 12 m 3 SV].

#### TPA perceived effectiveness of the intervention by the participants

Perceived effectiveness of the PREVENT program appeared to be associated with acceptability.

“*You could see the impact [on the performance data Hub] … I was surprised that just the fact of admitting [patients] and making sure that we got all of the testing done [made an impact].”* [101 12 m 5 SV].

*“..we changed the process and so it’s not something that I have to monitor every day … we changed the culture … the strength was that it did not depend on one person in general. I think that [our PREVENT program] showed that also simple changes could make a huge difference.”* [101 SUS 5 SV].

*At times, a team member questioned whether their efforts to improve their quality was associated with their quality performance scores. “It was good to see the trending of our data points but you know just based on the numbers that I saw, you know sometimes I had questions about whether or not the number for that month or that I saw for that month was accurately reflecting what was going on at our site.”* [105 12 m 5 SV].

#### TPA self-efficacy

Teams reported having a sense of accomplishment after participating in PREVENT and completing their action plans. This team accomplishment appeared to associate with perceived acceptability after completing participation in PREVENT as denoted by a team member who was initially frustrated at 6 months with team delays:

“… *But then really I guess in our graduation event, the two things I’m most pleased with because we set out at the beginning [team kickoff] that those were really going to be the most useful things to happen and that was the dashboard, identify the patient, and the checklist. … And I think that the team I had was great in terms of being excited about the process and feeling like it was important for patient care. …. And I think we are providing better care than we were by having done the process. And if for no other reason than just raising awareness a lot. You know, I think our primary care providers are more aware, ED is more aware, even our medicine house staff. Then our resident teams are more cooperative with showing up and seeing the patient as opposed to just giving some advice over the phone which they were often apt to do..”* [105 12 m 2 SV].

Furthermore, viewing positive performance also came with its own caveat. At times positive performance brought complacency among the team participants as demonstrated at one site. *“… when the team feels like the numbers look good, they’re not pushing, it’s not a priority to make any additional changes … So you don’t feel pushed to the next level because you’re pretty decent where we are [according to the data reported on the Hub and ranked nationally]*.” [106 SUS 1 SV].

## Discussion

### Interpretation

The QI clinical teams deemed the PREVENT quality improvement program as acceptable. Overall, the team members reported high satisfaction with the overall PREVENT program after completing 12 months of active implementation. Acceptability grew over time in active implementation. For some participants, perceptions of acceptability were discussed in the context of their local PREVENT achievements and original team goals accomplished at the end of active implementation. For the majority of the teams (5/6), the clinical champions rated their overall satisfaction higher than their respective supportive team members. However this is to be expected because the clinical champions were often the consumers of the PREVENT resources and tools compared to their multidisciplinary team members.

### Generalisability

Compared to previous research which has often characterized overall healthcare program acceptability [26, 27] by its stakeholders or included Likert scale ratings of specific program components [28, 29], our results contribute to the literature on implementation and on complex intervention evaluations by expanding upon the measurement of program acceptability and applying a multi-component acceptability construct, The Theoretical Framework of Acceptability [4]. Participants with the high satisfaction ratings reported positive affect towards the PREVENT intervention and its components, resources, and materials; perceived the amount of effort required to participate as difficult at times (i.e., perceived burden) but nonetheless persevered with program uptake; perceived the intervention as a good fit with their values (ethicality); demonstrated intervention coherence (i.e., understood the intervention and how it worked); dedicated their time and effort to this intervention as the opportunity costs; demonstrated strong perceived effectiveness of the PREVENT program at their local facility; and as the participants’ accomplished their planned team goals, we observed that their self-efficacy increased over time during active implementation. Employing a multifaceted construct to evaluate program acceptability is a potential strategy to minimize socially desirable responses by its stakeholders as they have a framework to articulate multiple aspects of acceptability.

The teams had access to a full-time external facilitator who provided guidance and answered questions about the PREVENT program throughout active implementation. Most of the clinical champions reported downloading the professional materials from the PREVENT program online HUB library and presented to their local clinical providers and staff with often a positive reception.

Another topic that permeated across the multiple levels of satisfaction was the access and use of data to inform the teams’ QI activities. PREVENT was the first QI program to systematically provide facilities with quality data on their acute transient ischemic attack care based on a formative evaluation of the VA national healthcare system [6, 8]. Team reviews of their baseline facility’s quality performance was ranked as the most useful component of the team kickoff meeting by participants across the teams. Because the teams were multidisciplinary across services in a healthcare facility, not all team members were aware that automated cerebrovascular quality data was a function of the PREVENT data Hub and not widely available. Thus, a third of the team members at the kickoff rated the review of their quality data as most useful. Moreover, a few participants questioned the pass/fail rates on the PREVENT quality metrics and this appeared to temper acceptability. However, most of these participants expressed that despite this debate, they still utilized the data and found the PREVENT program to be acceptable.

Finally, our data demonstrated a temporality effect on perceived acceptability. That is, the QI team participants’ acceptability grew over the course of engaging in active implementation over 12 months. To our knowledge, this study is the first to report temporality effects associated with program acceptability.

Moreover, we observed that our QI team participants would often discuss their acceptability in the context of their team’s accomplishments and goal achievements after 12 months of active implementation. For some participants (e.g., pharmacists) with previous QI experience, they often perceived PREVENT as highly acceptable early in active implementation at 6 months. For other participants who were delayed with team related issues, their acceptability improved over time as they achieved their action plans and reported an increase in self-efficacy. Future research may prospectively test these relationships among these concepts.

### Limitations

Our data collection occurred prospectively across program implementation; however, we queried participants retrospectively after their experiences with the program elements. We did not ask about anticipated acceptability prior to the intervention at baseline. Moreover, our data represents QI teams from the national VHA. Our results may not generalize to other healthcare systems. Finally, we did not feedback our results to our participants for “member checking” [30]. Nonetheless, our study is one of the few applications of the multi-component TFA in a complex intervention implemented in a real-world, national healthcare setting.

## Conclusions

Program acceptability varied by temporality and by champion role on QI team. Clinical team members reflected upon the seven components comprising the multi-construct, Theoretical Framework of Acceptability. A complex quality improvement program that fostered a high degree of flexibility in local adaptation and supported users with access to data, resources, and implementation strategies was deemed acceptable by quality improvement clinical teams implementing practice changes in a large, national healthcare organization.

## Supplementary Information

**Additional File 1.**

**Additional File 2.**

**Additional File 3.**

**Additional File 4.**

## Data Availability

These data must remain on Department of Veterans Affairs servers; A limited, de-identified data set may be available.

## Abbreviations

CoP: Community of Practice
EF: External Facilitation
IRB: Institutional Review Board
MRC: Medical Research Council
PREVENT: Protocol-guided Rapid Evaluation of Veterans Experiencing New Transient Neurologic Symptoms
QI: Quality Improvement
TFA: Theoretical Framework of Acceptability
TIA: Transient Ischemic Attack
VAMCS: Veterans Administration Medical Centers
VHA: Veterans Health Administration

## References

[1] Proctor E, Silmere H, Raghavan R (2011). Outcomes for implementation research: conceptual distinctions, measurement challenges, and research agenda. Admin Pol Ment Health.

[2] Moore GF, Audrey S, Barker M (2015). Process evaluation of complex interventions: Medical Research Council guidance. BMJ.

[3] Weiner BJ, Lewis CC, Stanick C (2017). Psychometric assessment of three newly developed implementation outcome measures. Implement Sci.

[4] Sekhon M, Cartwright M, Francis JJ. Acceptability of healthcare interventions: an overview of reviews and development of a theoretical framework. BMC Health Serv Res. 2017;17(1):88. 10.1186/s12913-017-2031-8. 10.1186/s12913-017-2031-8 PMC5267473 28126032

[5] Bravata DM, Myers LJ, Arling G (2018). Quality of Care for Veterans with Transient Ischemic Attack and Minor Stroke. JAMA Neurol.

[6] Damush TM, Miech EJ, Sico JJ (2017). Barriers and facilitators to provide quality TIA care in the veterans healthcare administration. Neurology.

[7] Homoya BJ, Damush TM, Sico JJ (2019). Uncertainty as a key influence in the decision to admit patients with transient ischemic attack. J Gen Intern Med.

[8] Bravata DM, Myers LJ, Homoya B (2019). The protocol-guided rapid evaluation of veterans experiencing new transient neurological symptoms (PREVENT) quality improvement program: rationale and methods. BMC Neurol.

[9] Bosworth HB, Olsen MK, Grubber JM (2009). Two self-management interventions to improve hypertension control: a randomized trial. Ann Intern Med.

[10] Bosworth HB, Powers BJ, Olsen MK (2011). Home blood pressure management and improved blood pressure control: results from a randomized controlled trial. Arch Intern Med.

[11] Damschroder LJ, Aron DC, Keith RE (2009). Fostering implementation of health services research findings into practice: a consolidated framework for advancing implementation science. Implement Sci.

[12] Ranta A, Dovey S, Weatherall M (2015). Cluster randomized controlled trial of TIA electronic decision support in primary care. Neurology.

[13] Rothwell PM, Giles MF, Chandratheva A (2007). Effect of urgent treatment of transient ischaemic attack and minor stroke on early recurrent stroke (EXPRESS study): a prospective population-based sequential comparison. Lancet.

[14] Damush TM, Miech EJ, Rattray NA (2021). Implementation Evaluation of a Complex Intervention to Improve Timeliness of Care for Veterans with Transient Ischemic Attack. J Gen Intern Med.

[15] Rattray NA, Damush TM, Miech EJ, et al. Empowering implementation teams with a learning health system approach: leveraging data to improve quality of care for transient ischemic attack. J Gen Intern Med. 2020. 10.1007/s11606-020-06160-y. 10.1007/s11606-020-06160-y PMC7652965 32875510

[16] Powell BJ, Waltz TJ, Chinman MJ (2015). A refined compilation of implementation strategies: results from the expert recommendations for implementing change (ERIC) project. Implement Sci.

[17] Rattray NA, Damush TM, Luckhurst C (2017). Prime movers: advanced practice professionals in the role of stroke coordinator. J Am Assoc Nurse Pract.

[18] Ritchie MJ, Kirchner JE, Townsend JC (2020). Time and organizational cost for facilitating implementation of primary care mental health integration. J Gen Intern Med.

[19] Cruess RL, Cruess SR, Steinert Y (2018). Medicine as a Community of Practice: implications for medical education. Acad Med.

[20] Penney LS, Homoya BJ, Damush TM, Rattray NA, Miech EJ, Myers LJ, Baird S, Cheatham A, Bravata DM (2021). J Gen Intern Med..

[21] Kizer KW, Dudley RA (2009). Extreme makeover: transformation of the veterans health care system. Annu Rev Public Health.

[22] Hagg HW, Workman-Germann J, Flanagan M, et al. Implementation of Systems Redesign: Approaches to Spread and Sustain Adoption. In: Henriksen K, et al., editors. Advances in Patient Safety: New Directions and Alternative Approaches (Vol. 2: Culture and Redesign). Rockville: Agency for Healthcare Research and Quality (US); 2008. 21249910

[23] Bravata DM, Myers LJ, Perkins AJ, Zhang Y, Miech EJ, Rattray NA, Penney LS, Levine DA, Sico JJ, Damush TM (2020). Effectiveness of the protocol-guided rapid evaluation of veterans experiencing new transient neurological symptoms (PREVENT) quality improvement program: a nonrandomized cluster trial. JAMA Netw Open.

[24] NVivo qualitative data analysis software. QSR International Pty Ltd. Version 12. 2018.

[25] Miles MBHA, Saldana J (2014). Qualitative data analysis: a methods sourcebook.

[26] Damron-Rodriguez J, White-Kazemipour W, Washington D (2004). Accessibility and acceptability of the Department of Veteran Affairs health care: diverse veterans' perspectives. Mil Med.

[27] Fulop N, Boaden R, Hunter R (2013). Innovations in major system reconfiguration in England: a study of the effectiveness, acceptability and processes of implementation of two models of stroke care. Implement Sci.

[28] Mealer M, Conrad D, Evans J (2014). Feasibility and acceptability of a resilience training program for intensive care unit nurses. Am J Crit Care.

[29] Paladino J, Bernacki R, Neville BA (2019). Evaluating an intervention to improve communication between oncology clinicians and patients with life-limiting Cancer: a cluster randomized clinical trial of the serious illness care program. JAMA Oncol.

[30] S. LY, E.G. G (1985). Naturalistic Inquiry.

