# Supplementary material for: Acceptability of a complex team-based quality improvement intervention for transient ischemic attack: a mixed-methods study
Source: BMC Health Serv Res. 2021 May 12;21:453. doi: 10.1186/s12913-021-06318-2 (PMC8117601; doi:10.1186/s12913-021-06318-2)
Supplement: Supplementary file 1 — Additional File 1. [file 12913_2021_6318_MOESM1_ESM.pdf]

PREVENT 6 month Semi-Structured Interview Guide

Verbal informed consent per IRB: Briefly explain the purpose of the interview to the participant per the Study Information form and obtain verbal informed consent. Consent is implied by participation.

Please read the following statement to the participant: **All responses are confidential and voluntary. Individual responses will not be shared with management.**

[START HERE FOR ALL PARTICIPANTS]<br>"This is an interview with [PARTICIPANT NAME] on [DATE].<br>To begin, let's start with what is your position and job title?<br>How long have you worked at the [facility name] VA medical center?<br>How does the work that you do at your facility relate to providing care for Veterans with TIA?"

We are visiting your facility today to ask you and your colleagues about the current state of your TIA care processes as well as questions about how the facility and more specifically how clinicians are organized to improve quality of TIA care. In addition, we are interested in your perspective on the local PREVENT program and its implementation at your facility over the last six months.

**1**	**Acute TIA Care**
**1a**	Does your facility offer acute TIA care 24/7? Or during limited hours only – M-F 8-5?

**2**	**PREVENT Program Perspective**
**2a**	How have you been involved so far with PREVENT at your facility?
**2b**	From your perspective, in general how is the implementation of the local PREVENT plan going?
**2c**	What would you say are the strengths of how the program has been implemented so far?
**2d**	What barriers have you and/or the team faced during making local process changes for TIA care?
**2e**	What activities have you yourself participated in terms of implementing the local PREVENT program?
**2f**	At the kick off, your team made an action plan which included X, Y, Z. How much progress do you think your team has made on this action plan?
**2g**	-What do you think might explain this level of progress so far?
**2h**	-What are your thoughts about the PREVENT program? [prompts: strength of evidence, appropriateness, relative advantage, complexity].

3 Kickoff

3a Did you attend the kick off?

3b Looking back, what was the most helpful part of the kick off for you in terms of improving the quality of TIA care - why?

3c What was the least helpful - why?

3d Thinking back, was there anything else that would have better prepared you to adopt PREVENT locally?

3e How important was the kick-off in forming a local team of providers for TIA care?

4 Hub

Usage/Experience:

4a Have you visited the PREVENT data HUB on the internet?

4b -Why not [if no]?

4c -If yes, what section(s) have been most helpful/least helpful? Why?

4d -Any places you have not visited yet?

4e When was the last time that you visited the Hub?

Data:

4f What are your thoughts on how the performance data is presented?

4g What do you think of the "WITHOUT FAIL RATE"?

4h Do you think that rate reflects the quality of your care that you and your colleagues are providing at your facility? Why or why not?

4i What do you think about seeing the arrow pointed up or down on your facility's performance?

Library/Planning:

4j Have you used any of the library materials? Which? Why? How useful were they?

4k Have you or your team modified materials for local use?

4l How often did you look at your team's plan on the HUB?

4m Did you personally make any changes to your team's plan on the HUB?

4n How often did you look at other facility's plans?

5 Data

Some facilities in the PREVENT program have requested that our team provide patient level data for patients with TIA at their facility, including TIA patient lists, pass rates for each of the 7 without fail measures, and 90day mortality and recurrent event rates

5a Did you make such a request?

-If yes, how did you use the data that was provided? {Probe to see if it was used for data validation/verification? To understand patterns in pass rates? }

-If not, would it be helpful for you to see data like this? How would you use the data?

5b Would you prefer to see another type of report? What specifically?

5c How frequently would you like to see your facility data? {If needed, remind them that data is updated monthly on the HUB.}

5d Do you trust the data you see displayed on the HUB?

On a scale from 1-10 where 1 indicates "not at all" and 10 indicates "a high level of trust", how much do you trust the data?

5e Which data in the Hub do you have the most confidence? The least confidence?

5f Are there data from the VA that you trust more?

5g Do you personally ever receive data about any kind of quality of care at your facility? <u>If yes:</u>

5h What do they look like? Content, mode, form? How helpful are those reports? How can they be improved?

5i How representative of your performance are those reports?

5j How do you prefer data reports relevant to you to be delivered? (Electronically? In paper format? Excel spreadsheet?) Why?

5k If you could have the data delivered to you in any format, what design would motivate you the most to take the time to review its contents? To act upon its contents?

5l Ideally, what data would be the most important for you to receive about your performance?

5m What data might motivate you to change your performance?

5n How frequently would you want to see this data? What mode of delivery would be best for you to receive this data? Who else, if anyone, would you want to see your performance feedback? Why?

5o Do you have a preference for running data reports in Vista? In CDW?

6 Quality Performance Comparisons

One of the HUB features is your ability to compare your facility's performance to other VAMCs.
6a How often did you or your team make this comparison?

6b [If yes], which VAMC[s] did you compare your facility?
6c Why those?
6d What did you think after viewing other facilities' performance?

7 Collaborative Calls

7a How often have you attended the monthly calls?

7b What information from those calls has been the most helpful to you? Least helpful? Why?

7c What are your thoughts about hearing updates from other facilities?

Have you been able to adapt any of the other teams' protocols or materials to your local
7d PREVENT program?
7e If so, which and how did it turn out?

Do you have any future plans to implement projects or protocols that have been developed by
7f other VAMCs in PREVENT?

Have you had direct communications with another PREVENT team member from another VA
7g facility?
7h If so, Who? How often? What motivated you to reach out?

7i Have other PREVENT teams reached out to you?
7j If so, what was the reason?

Are you involved in any cerebrovascular professional organization? Any other VA collaborative
7k – Emergency Medicine, Pharmacy, Nursing?
7l If so, how often does the group meeting and by what mode (Virtual, Telephone, In-person?)

7m How much do you feel part of a community of practice for TIA care?
[prompt: Do they think they have gotten to know the PREVENT participants/staff?
More so than a general listserv?]

8 Communication/Access to RN Facilitator

8a How often during the past 6 months did you contact Ms. Barbara Homoya to discuss PREVENT either directly by telephone or through email to her?

8b What were the reasons for the direct discussions or the topics discussed?

8c How helpful was it, if at all, for you to have direct access to the PREVENT facilitator?

8d Do you anticipate any future needs in terms of facilitation from Ms. Homoya or the PREVENT team in Indianapolis?

8e How often did you or your facility receive CAC expertise from Indianapolis? Other?

9 Shared PREVENT Materials

9a Were you able to use any of the existing PREVENT materials provided by the national program?

9b -Which ones?

9c -How did you adapt to your local facility?

9d How helpful was it for your team to have access to the shared PREVENT materials?

9e How often did you share your PREVENT materials and program with your peers at your facility?

9f What has been the reaction of your peers to the PREVENT program at your facility?

10 Training

10a What was your strategy for training your local staff on the PREVENT program and materials?

10b Did you make any changes along the way during the past 6 months?

10c -What changes were those?

10d Were there any strategies for sustaining a training program to address new staff and provider turnover?

11 Motivation to Participate

11a What would you say is the most important reason for why you work on TIA processes of care?

11b -With your local team?

11c How much do you like participating in PREVENT?

11d How prepared do you feel to implement PREVENT and to contribute to care of TIA patients (self-efficacy)?

**12**	**Team Activation**
**12a**	How did the PREVENT team at your facility materialize?
**12b**	Who formed the team – that is, who invited others? [Person(s); Snowball]
**12c**	How does the team usually communicate with each other and how often?
**12d**	Does the team interact with others at your facility for the purpose of PREVENT? Or other reason?
**13**	**Goals**
**13a**	Did your local PREVENT team set a team goal? If yes:
**13b**	What was it?
**13c**	How was the goal chosen?
**13d**	How were the goals evaluated?
**13e**	Were there subgoals set up?
**13f**	How were the team members accountable to the team in achieving their tasks?
**14**	**Plans**
**14a**	How did your local PREVENT team make plans over time?
**14b**	Who set the plans?
**14c**	Who tracked the progress?
**14d**	How formal was your team's process for planning?
**14e**	Did your team allocate time specifically for planning?

15 **Reflecting and Evaluating**

One of the features of the PREVENT program is the availability of your facility's quality performance data.

15a How did the PREVENT team at your facility use data from the HUB?

15b What decisions were made based upon the data on the HUB?

15c Which team members generally accessed the HUB quality performance data for your team?

15d What did your team do when your local team's performance was shown to improve? Decline?

15e Did you compare your team's performance to other VA facilities? If so,

15f -Which ones?

15g -Why those?

15h -How did you performance compare?

16 **Local adaptation**

[We will use the PREVENT document which depicts the site's local PREVENT adaptation. We won't ask about this concept but the participant may discuss].

17 **Champions**

17a Looking back at the past 6 months, How has the PREVENT program helped your local champion (the site lead for PREVENT)?

17b What activities by your local site lead stand out with you as important for the local PREVENT implementation?

17c What role(s) did the Pharmacists at your facility play in your local PREVENT implementation?

17d Others – [IF a particular service provides a good amount of care for TIA, then please ask about that service here]

18 Other Implementation Outcomes

18a How widely has the local PREVENT program been adopted; what has been the uptake by local providers?

18b Has the program been implemented as intended (fidelity)? In everyday practice, how suitable is the program at your VAMC?

18c In terms of how widely the program has been adopted, roughly what percentage of providers who could adopt the program are currently implementing it?

18d Are they adopting consistently?

18e What do you see as key facilitators or barriers to making the program *sustainable* at your VAMC?

18f Are there any outside forces affecting your implementation efforts here at your facility?

LOCAL CONTEXT RELATED TO TIA AND STROKE

19 Policy

19a Does your facility have a written protocol or pathway for patients with suspected TIA?
If yes, please describe. [If only a stroke protocol, clarify that they do not have a specific TIA protocol]

19b If yes, where is it posted or kept?

19c How urgent do you view TIA care is now? [compared to prior PREVENT participation].

20 Leadership engagement

20a How has the local PREVENT team engaged with leadership [at level of service, facility, visn, regional, national]?

20b Does anyone from PREVENT team report to the Facility Director?

20c Are meeting notes submitted to the facility/VISN?

20d How might implementation of a TIA program align with other organizational goals?
[prompt: for example, might it align with facility goals for risk factor management?]

20e How important is the implementation of a TIA program to your organization’s Executive Leadership? Why?

21 Use of Data at Local PREVENT Program

21a Did you generate your own data?

21b What was the frequency with which you reviewed the process of care data on the hub?

21c Have you or the team corporated process of care data into routine work flow?

21d Did you or your colleagues conduct your own chart reviews?

21e Did the collaborative calls increase your utilization of the hub?

21f Did the calls create a sense of professional community?

21g Did the calls serve to maintain your interest and enthusiasm about TIA quality improvement?

22 Risk Score

As part of the PREVENT program, participating sites are provided with their patient risk score which is a measure of the risk of 1-year mortality. We are interested in two analyses related to the risk score.

22a How did the sites use the patient risk score?

22b What was the single most important element of PREVENT participation for you working in your facility: kickoff, monthly process data on the hub, having access to the PREVENT community, the monthly calls, the library of resources and materials on the hub?

**23**	**General Questions**
**23a**	What are some of the strengths that you see in the current set-up for acute TIA care?
**23b**	What barriers do you see to providing TIA care at this facility?
**23c**	How do you think additional resources might be directed toward TIA patients at this site?
**23d**	What other observations or comments do you have to share with us about current TIA care coordination at this facility?
**23e**	Have you been involved in any planning activities for your service area during the past year at your facility? [prompt: May stroke awareness month; Plans for OIG visit; Practice change?]
**23f**	Tell us what was it specifically for and who were involved. How well do you think your facility executed against those plans?
**23g**	Have you participated in any virtual/online professional community during the past year
	If yes,
**23h**	-Which communities?
**23i**	-What did you like about those?
**23j**	-What did you not like?
**23k**	Ideally, what do you think are essential components to a thriving virtual community and learning collaborative?
