# Supplementary material for: Acceptability of a complex team-based quality improvement intervention for transient ischemic attack: a mixed-methods study
Source: BMC Health Serv Res. 2021 May 12;21:453. doi: 10.1186/s12913-021-06318-2 (PMC8117601; doi:10.1186/s12913-021-06318-2)
Supplement: Supplementary file 2 — Additional File 2. [file 12913_2021_6318_MOESM2_ESM.pdf]

PREVENT 12 MONTH Semi-Structured Interview Guide

**Interviewer:**	**Date:**
**Facility:**	**Participant:**
**VISN:**	**Job Title:**
**Interview #**	

INSTRUCTIONS:
Verbal informed consent per IRB: Briefly explain the purpose of the interview to the participant per the Study Information form and obtain verbal informed consent. Consent is implied by participation.

Please read the following statement to the participant: **All responses are confidential and voluntary. Individual responses will not be shared with management.**

[START HERE FOR ALL PARTICIPANTS]<br>"This is an interview with [PARTICIPANT NAME] on [DATE].<br>To begin, let's start with what is your position and job title?<br>How long have you worked at the [facility name] VA medical center?<br>How does the work that you do at your facility relate to providing care for Veterans with TIA?"

We are visiting your facility today to ask you and your colleagues about the current state of your TIA care processes as well as questions about how the facility and more specifically how clinicians are organized to improve quality of TIA care. In addition, we are interested in your perspective on the local PREVENT program and its implementation at your facility over the last 12 months.

First, let's begin with acute TIA care at your facility. Currently, does your facility offer acute TIA care 24/7? During limited hours only – M-F 8-5? Has anything changed in the last 12 months?

PREVENT Program Perspective

- How have you been involved with PREVENT at your facility over the past year? When did you join the team working at your facility, and how did you find out about it?
-
- What would you say are the strengths of how the program has been implemented over the year?
- What barriers have you and/or the team faced during making improvements in TIA care? How did you overcome these barriers? Could you give an example or two?
- What activities have you yourself participated in terms of implementing the local PREVENT program?

From your perspective, in general how well was the implementation of the local PREVENT plan executed? At the kick off, your team made an action plan which included X, Y, Z. How much progress do you think your team has made on this action plan?

- What do you think might explain this level of progress over the year?
- What are your thoughts about the PREVENT program? [prompts: strength of evidence, appropriateness, relative advantage, complexity].

1. How well has PREVENT spread across the front-line staff? [adoption]
2. In terms of how widely the program has been adopted, roughly what percentage of providers who could adopt the program are currently implementing it? Are they adopting consistently? What do you see as key facilitators or barriers to making the program *sustainable* at your VAMC?
3. Are there any outside forces affecting your implementation efforts here at your facility?

4. How confident are you that PREVENT will be sustained at your local VAMC? Why or why not?
5. What local resources have been secured or provided for PREVENT?
6. What additional resources are still needed to sustain PREVENT at your facility?
7. What tools and components of PREVENT do you think were the most helpful to you and your colleagues? What were the least? Why?

<u>HUB=</u>

- Now I'd like to ask you about the PREVENT Data Hub.
- First, did you receive one on one training from Barb Homoya, our facilitator? What did you learn about the Hub? [Prompt: If cannot recall, do you think you were able to navigate better after the individual meeting?].
- Have you attended any of the Hub Office Hours that Prevent has offered? [If no, skip to next question.] If yes, why did you decide to attend the Hub Office Hours? What did you wish to learn about the Hub? Did attending the Hub Office Hours help you to navigate the Hub better? Did attending help you to find specific information on the Hub? [If yes, what information?]
- At any time, did you ask the PREVENT team about the definitions or specifications of the process measures [the processes that comprise the Without Fail Rate]? If yes, how satisfied were you with the responses you received to your questions? How satisfied were you to the changes the PREVENT team made to the measurement specifications based on your questions/concerns?
- On several occasions, Dr. Laura Myers, PREVENT's Data Scientist, made presentations that demonstrated how the process measures were defined and evaluated. How helpful or not, were these presentations for your understanding of the PREVENT process outcomes?
- DURING THE PAST 6 MONTHS (Months 6-12)
- Usage/Experience: When was the last time that you visited the HUB? Have you visited the PREVENT data HUB on the internet recently? Why not [if no]? If yes, what section(s) have been most helpful? Why? Least helpful or places you did not visit yet – why?
- Data: What are your thoughts on how the performance data is presented? What do you think of the "WITHOUT FAIL RATE"? Do you think that rate reflects the quality of your care that you and your colleagues are providing at your facility? Why or why not? What do you think about seeing the arrow pointed up or down on your facility's performance?

- Library/Planning: Have you used any of the library materials? Which? Why? How useful were they? Have you or your team modified materials for local use? How often did you look at your team's plan on the HUB? Did you personally make any changes to your team's plan on the HUB? How often did you look at other facility's plans?
- Some facilities in the PREVENT program have requested that our team provide patient level data for patients with TIA at their facility, including TIA patient lists, pass rates for each of the 7 without fail measures, and 90day mortality and recurrent event rates
- Did you make such a request? If yes, how did you use the data that was provided? {Probe to see if it was used for data validation/verification? To understand patterns in pass rates? }
- If not, would it be helpful for you to see data like this? How would you use the data?
- Would you prefer to see another type of report? What specifically?
- How frequently would you like to see your facility data? {If needed, remind them that data is updated monthly on the HUB.}
- Do you trust the data you see displayed on the HUB? On a scale from 1-10 where 1 indicates "not at all" and 10 indicates "a high level of trust", how much do you trust the data? Which data in the Hub do you have the most confidence? The least confidence?
- Are there other data from the VA that you trust more?

Do you personally ever receive data about any kind of quality of care at your facility? If yes:

3a ☐ What do they look like? Content, mode, form?

3b ☐ How helpful are those reports?

3c ☐ How representative of your performance are those reports?

3d ☐ How can they be improved?

3e ☐ How do you prefer data reports relevant to you to be delivered? (Electronically? In paper format? Excel spreadsheet?) Why?

3f ☐ If you could have the data delivered to you in any format, what design would motivate you the most to take the time to review its contents? To act upon its contents?

3g ☐ Ideally, what data would be the most important for you to receive about your performance? What data might motivate you to change your performance?

3h ☐ How frequently would you want to see this data? What mode of delivery would be best for you to receive this data? Who else, if anyone, would you want to see your performance feedback? Why?

3i ☐ *IF RELEVANT:*

Do you have a preference for running data reports in Vista? In CDW?

[Some sites do not use CDW at all because the VISN will not give access].

RISK SCORE: Did you view the patient risk score for your facility on the HUB? If no, why not? If yes, what did you think about that score? Do you think it accurately reflects your patient risk? How did you or your colleagues use the patient risk score on the HUB?

Quality Performance Comparisons: One of the HUB features is your ability to compare your facility's performance to other VAMCs. How often did you or your team make this comparison? [If yes], which VAMC[s] did you compare your facility? Why those? What did you think after viewing other facilities' performance? Were you primarily interested in the Without Fail Rate, or were there specific measures for which you found comparison to national data or other facilities was most useful?

COLLABORATIVE CALLS – DURING THE PAST 6 MONTHS: How often have you attended the monthly calls? What information from those calls has been the most helpful to you? Least helpful? Why: could you give an example?
What are your thoughts about hearing updates from other facilities? Have you been able to adapt any of the other teams' protocols or materials to your local PREVENT program? If so, which and how did it turn out? Do you have any future plans to implement projects or protocols that have been developed by other VAMCs participating in PREVENT?

- Have you had direct communications with another PREVENT team member from another VA facility? Who? How often? What motivated you to reach out?
- Have other PREVENT teams reached out to you? If so, what was the reason?
- Are you involved in any cerebrovascular professional organization? Any other VA collaborative – Emergency Medicine, Pharmacy, Nursing? If so, how often does the group meeting and by what mode (Virtual, Telephone, In person meeting)?
- How much do you feel part of a community of practice for TIA care? [prompt: Do they think they have gotten to know the PREVENT participants/staff? More so than a general listserv?

- Did the calls increase your utilization of the hub?
- Did the calls create a sense of profession community?
- Did the calls serve to maintain your interest and enthusiasm about TIA quality improvement?
- What was the single most important element of PREVENT participation for you working in your facility: kickoff, monthly process data on the hub, having access to the PREVENT community, the monthly calls, the library of resources and materials on the hub?

Communication/Access to RN facilitator= How often during the past 6 months did you contact Ms. Barbara Homoya to discuss PREVENT either directly by telephone or through email or Instant Messaging to her? What were the reasons for the direct discussions or the topics discussed? How helpful was it, if at all, for you to have direct access to the PREVENT RN facilitator?
Communication/Access to MD facilitator= How often during the past 12 months did you contact Dr. Dawn Bravata to discuss PREVENT either directly by telephone or through email or by Instant Messaging to her? What were the reasons for the direct discussions or the topics

discussed? How helpful was it, if at all, for you to have direct access to the PREVENT MD facilitator?
Do you anticipate any future needs in terms of facilitation from Ms. Homoya, Dr. Bravata or the PREVENT team in Indianapolis?

How often did you or your facility receive CAC expertise from Indianapolis? Other?

Shared PREVENT Materials=Were you able to use any of the existing PREVENT materials provided by the national program? Which ones? How did you adapt to your local facility? How helpful was it for your team to have access to the shared PREVENT materials? How often did you share your PREVENT materials and program with your peers at your facility? What has been the reaction of your peers to the PREVENT program at your facility?

Training – What was your strategy for training your local staff on the PREVENT program and materials? Did you make any changes along the way during the past 6 months? What changes were those? Were there any strategies for sustaining a training program to address new staff and provider turnover?

Motivation to participate: What would you say motivates you to participate in PREVENT? Has this changed over the course of the program – why? How prepared do you feel to sustain PREVENT and to contribute to care of TIA patients (self-efficacy)?

Team Activation:
Reflecting and Evaluating – One of the features of the PREVENT program is the availability of your facility's quality performance data. DURING THE PAST 6 MONTHS, How did the PREVENT team at your facility use data from the HUB? [prompt for how they may have used the data].

- What decisions were made based upon the data on the HUB?
- Which team members generally accessed the HUB quality performance data for your team?
- What did your team do when your local team's performance was shown to improve? Decline?
- Did you compare your team's performance to other VA facilities? If so, which ones? Why those? How did you performance compare?

Goals – Did your local PREVENT team set one or more team goal(s)? If yes, what was it?

- How was the goal chosen and was it modified over time?
- How were the goals evaluated? Were there subgoals set up?
- How were the team members accountable to the team in achieving their tasks?

Plans – How did your local PREVENT team make plans over time?

- Who set the plans?
- Who tracked the plan progress?
- How formal was your team's process for planning?
- Did your team allocate time specifically for planning?

Local Adaptation: [In addition to the site's local PREVENT adaptation figures. How has PREVENT been adapted over the course of the year at your facility (if at all)? That is what changes to the local PREVENT program elements have been made? Are there any unique aspects to your facility or context that played a role in how PREVENT was implemented in your medical center?

CHAMPIONS: Looking back at the past 6 months, **How has the PREVENT program helped your local champion (the site lead for PREVENT)? What activities by your local site lead stand out with you as important for the local PREVENT implementation?**

What role(s) did the Pharmacists at your facility play in your local PREVENT implementation?

-Others – [IF a particular service provides a good amount of care for TIA, then please ask about that service here.

Have any other team members emerged as TIA champions? Who? Why do you think so?

Local Context Related to TIA and Stroke

POLICY

Does your facility have a written protocol or pathway for patients with suspected TIA? If yes, please describe. [If only a stroke protocol, clarify that they do not have a specific TIA protocol]. If yes, where is it posted or kept? Can you describe how it was developed during the yearlong implementation phase?

How urgent do you view TIA care is now? [compared to prior PREVENT participation].

Leadership engagement- How has the local PREVENT team engaged with leadership [at level of service, facility, visn, regional, national]? Does anyone from PREVENT team report to the Facility Director? Are meeting notes submitted to the facility/VISN?

How might implementation of a TIA program align with other organizational goals?

PROMPT: For example, might it align with facility goals for risk factor management?

How important is the implementation of a TIA program to your organization's Executive Leadership? Why?

GENERAL QUESTIONS:

1. Thinking about future PREVENT implementation, what advice would you give to personnel at a VAMC who wants to implement PREVENT?
2. Overall, on a 1-7 scale where 1 indicates "not at all" and a 7 indicates "highly satisfied", how satisfied were you with the PREVENT program? Why?

Looking back, what would you have done differently if you knew then what you know now? How do you think your team would have operated differently if you had another opportunity like this?

In terms of the implementation activities you have undertaken, which have been the most efficient? Which of your activities has been high impact? Low or high effort?

What are some initiatives or future plans for your service and/or this facility in the next year? Have you been involved in any planning activities for your service area during the past year at your facility?

What other observations or comments do you have to share with us about current TIA care coordination at this facility?

Have you participated in any virtual/online professional community during the past year? (If yes, which communities?) What did you like about those? What did you not like? Ideally, what do you think are essential components to a thriving virtual community and learning collaborative
