# Supplementary material for: Acceptability of a complex team-based quality improvement intervention for transient ischemic attack: a mixed-methods study
Source: BMC Health Serv Res. 2021 May 12;21:453. doi: 10.1186/s12913-021-06318-2 (PMC8117601; doi:10.1186/s12913-021-06318-2)
Supplement: Supplementary file 3 — Additional File 3. [file 12913_2021_6318_MOESM3_ESM.pdf]

PREVENT Semi-Structured Interview Guide – Sustainability

**Interviewer:**	**Date:**
**Facility:**	**Participant:**
**VISN:**	**Job Title:**
**Interview #**	**Kickoff:**
	**Active Implementation:**
	**Sustainability Phase:**

INSTRUCTIONS:

[Note: For any new participant defined as a site employee who had not previously completed a baseline, 6 or 12 month interview, please obtain verbal informed consent per IRB: Briefly explain the purpose of the interview to the participant per the Study Information Sheet and obtain verbal informed consent. Consent is implied by participation.]

We are conducting this telephone interview with you today as a follow up since your local PREVENT team completed its active implementation on [*insert date of graduation from active implementation*]. We are interested in specific activities devoted to sustaining your local PREVENT program as well as the current state of your TIA care processes, any new initiatives, as well as questions about how the facility and more specifically how clinicians are organized to continue to improve the quality of TIA care.

[*Please read the following statement to the participant*:] **All responses are confidential and voluntary. Individual responses will not be shared with management. We plan to aggregate the individual data.**

- "This is an interview with [PARTICIPANT NAME] on [DATE].
- To begin, please tell me your position here at the VA and your job title?
- How long have you worked at the [facility name] VA medical center?
- How does the work that you do at your facility relate to providing care for Veterans with TIA?"

Commented [RNA1]: involved in? Devoted has a strong connotation.

Commented [PLS2]:

Commented [PLS3]: Do we need to ask this of ppl who have been interviewed before? If the interviewee has already provided this in the past, maybe instead ask, "What if any changes have occurred to your role since we last spoke?"

Commented [HB4R3]: Agree and like Lauren's question

GENERAL PREVENT PROGRAM PERSPECTIVE

- How have you been involved with PREVENT program at your facility? Since your local team graduated from active implementation of PREVENT, what has been your role with the local PREVENT program?
  - [If new participant]..When did you join the team working at your facility?
  - [If new participant] How did you find out about it?
- In terms of implementing the local PREVENT program at your facility, what are the activities in which you assisted?
- [*Note to interviewer*: *After giving the participant a chance to talk about their local PREVENT program*, *look at the team's goal achievement and accomplishments from the active implementation list of achievements at graduation found in the collaborative calls subfolder on PREVENT folder on Q drive and their rate of Goal attainment from the kickoff….]*
- At the kick off, your team made an action plan which included ~~X, Y, Z~~[*Note: fill in list from action plan*]. At graduation from active implementation, your team had completed [*Note: fill in list from graduation achievement list*]~~X, Y, Z~~ from your team's action plans.
  - Did your team make further progress on this initial action plan after graduation? If yes, what specifically? [If no, why not?]
  - What do you think might explain this level of progress after active implementation?
  - Did your team make plans for any new goals after graduation from active implementation? If yes, how were those new goals chosen? What were they? How much progress has your team made on these new goals?
- How often since graduation, has your local PREVENT team met to review its local performance? How often has your local PREVENT team made plans to change practice based on its TIA performance data since graduation?
- From your perspective, how well was the implementation of the local PREVENT plan executed?
- What are your thoughts about the PREVENT program overall? [prompts: strength of evidence, appropriateness, relative advantage, complexity].
- How well has PREVENT or protocols related to TIA spread or been disseminated across the front-line staff? [adoption] How do you know whether PREVENT has spread? [Prompt: Have you looked at any medical records; data; staff discussions specifically on implementing the PREVENT local program].
- In terms of how widely the program has been adopted, roughly what percentage of clinical providers and staff who could adopt the program are currently implementing it at

Commented [PLS5]: Might be good reorganize and try to streamline this section. Maybe have them give some updates on what's going on with their role and the local implementation team. Then detailed questions about goals, activities/adaptations, challenges and successes since active implementation. Then move into more reflective questions about what they think about their local implementation and the PREVENT program overall.

Commented [PLS6]: Again, to reduce respondent burden, would suggest not asking this of people who have been interviewed multiple times in the past. For longitudinal interviews, I'll often paraphrase information that they've provided in past and ask if that's correct and/or for updates.

Commented [PLS7]: Do we need a question that asks about the status of the local PREVENT program? Like, are they meeting? Regularly or ad hoc basis? Is it still an initiative/something being worked on or has it transitioned to standard practice?

Commented [PLS8]: I think people will probably also note why they became involved – but just in case they don't, do we want a probe into why? I think it'd be interesting to know if people opt into participating because they had good experiences with it, or whether new people are being tasked to participate (possibly because others are being pulled away/opting out).

Commented [PLS9]: Potentially repetitive of the first question? Also, do we want to specifically know what they've been doing post-active implementation?

Commented [PLS10]: Is the interviewer doing this with the interviewee or on their own? If on their own, seems like prep work prior to interview – so perhaps there should be a note at the beginning to reminder interviewer to tailor interview guide to the site prior to the interview?

Formatted: Font: Italic

Formatted: Font: Italic

Commented [PLS11]: There's a lot of questions here, maybe break out into a main question and subquestions?

Commented [PLS12]: This seems potentially repetitive of the question about abt whether they've created new goals since the end of active implementation. Maybe they could be combined or at least adjacent to one another?

Formatted: Font: (Default) Arial

Commented [PLS13]: Will people know what this means? Do we need to specify new processes put in place for TIA care and/or understanding of best practices for TIA patients?

Commented [PLS14]: Again, unsure whether people will understand what this means, it seems abstract.

your VA facility? [Prompts: ED, Hospitalists, Pharmacists, Neurologists, Residents, Radiologists, Primary Care, Nursing]

- Are they adopting consistently? How do you know?
- What do you see as key factors facilitators to making the PREVENT program *sustainable* at your VAMC?
- What do you see as keychallenges barriers to making the PREVENT program *sustainable* at your VAMC?

- Are there any outside forces affecting your implementation efforts here at your facility? [Prompts: National, or VISN, policies or local university affiliate policies; organizational priorities or resource issues]
- How has your local leadership supported your local PREVENT program, if at all?
- Since graduation from active implementation, what has been the strength of your local PREVENT program sustainment? Why do you think so?
- Do you think the local PREVENT team champion(s) is/are still actively sustaining your local PREVENT program? Why or why not?
- ~~How often since graduation, has your local PREVENT team met to review its local performance? How often has your local PREVENT team made plans to change practice based on its TIA performance data since graduation?~~
- What has been your local PREVENT team's greatest successes? Why do you think so?
- What barriers have you and/or the team faced while making improvements in TIA care since you graduated from active implementation?
  - How did you overcome these barriers?
  - Could you give an example or two?
- What TIA processes if any, haves been de-implemented – were any of the processes extinguished among the front line – what, how, why?
- How well has PREVENT worked for your facility following the implementation phase of the program? [after graduation on X date]
- How confident are you that PREVENT will be sustained at your local VAMC? ~~Why or why not?~~What factors do you think would play a role in sustaining existing efforts?
- What local resources have been secured or provided for PREVENT?
- What additional resources are still needed to sustain PREVENT at your facility?

Commented [PLS15]: What kind of nursing? IP, PACT, neuro…

Commented [PLS16]: Factors? I've been advised in the past that "facilitators" and "barriers" is jargony.

Commented [PLS17]: Challenges?

Commented [PLS18]: I think some sites might identify multiple champions

Commented [PLS19]: I think if there's a general question about the status of the local PREVENT team at the top, this could be a subquestion for that.

Commented [PLS20]: I would move these up to be with the questions about what's been going on locally since active impl

HUB

Now I'd like to ask you about the PREVENT Data Hub.

- When was the last time that you visited the HUB?
  - What section(s) have been most helpful? Why?
  - Least helpful or places you did not visit yet – why?
  - Are you familiar with the current data on the HUB?
- ~~Within~~ During the sustainability phase of the PREVENT Program, how often did you and the team review data from the HUB?
- What are your thoughts about the performance data that was presented at our final collaborative call (all sites increased their quality of care)? Were the data surprising or expected?
- What are your thoughts about the performance data that is currently presented?
  - How well do you think your local VAMC is providing ongoing TIA care?
  - What are the areas of greatest success in providing TIA care?
  - What are the areas of greatest struggle in providing TIA care?
  - What do you think of your local PREVENT team's "WITHOUT FAIL RATE"? (going up or down in the sustainability phase)
  - Do you think that rate reflects the quality of your care that you and your colleagues are providing at your facility? Why or why not?
- The HUB offers a location to enter a plan for improving TIA care. Has your team set any new goals in the last few months that were or could be entered into the HUB?
- Were there any plans made and entered onto the HUB for the sustainability phase?

Commented [PLS21]: Repeats question above.

Commented [PLS22]: Will everyone interviewed have attended? Will they all recognize which call referred to? Do we need probes here? Like, did it surprise you? Did you believe it?

Commented [PLS23]: Unclear – maybe start instead with the first bullet?

Commented [PLS24]: Is struggle potentially too strong of a word? Challenge instead?

Commented [PLS25]: Repeats above. Maybe instead ask whether since active impl their team has updated any of their materials on the HUB?

Quality Performance Comparisons:

One of the HUB features is your ability to compare your facility's performance to other VAMCs.

- How often did you or your team make this comparison during the sustainability?
  - [If yes], with which VAMC[s] did you compare your facility? Why those?
  - What did you think after viewing other facilities' performance?

- Were you primarily interested in the Without Fail Rate, or were there specific measures for which you found comparison to national data or other facilities was most useful? What is your perspective on how the Without Fail Rate can be used to improve TIA care?

COMMUNICATION/ACCESS TO RN & MD FACILITATORS

- How often during the sustainability phase of PREVENT did you contact Ms. Barbara Homoya to discuss PREVENT either directly by telephone or through email or Instant Messaging?
  - What were the reasons for the direct discussions or the topics discussed?
  - How helpful was it, if at all, for you to have direct access to the PREVENT RN facilitator?
- How often during the sustainability phase of PREVENT did you contact Dr. Dawn Bravata to discuss PREVENT either directly by telephone or through email or by Instant Messaging?
  - What were the reasons for the direct discussions or the topics discussed?
  - How helpful was it, if at all, for you to have direct access to the PREVENT MD facilitator?
- Do you anticipate any future needs in terms of facilitation (whether from Ms. Homoya, Dr. Bravata, or others) -from or the PREVENT team in Indianapolis?

Commented [HB26]: Just a reminder that I won't be available after Sept 28. Dawn and I are discussing when/how to advise the site participants of this.

SHARED PREVENT MATERIALS

- Were you able to use any of the existing PREVENT materials provided by the national program during the sustainability phase? Which ones?
  - How did you adapt to your local facility?
  - How helpful was it for your team to have access to the shared PREVENT materials?
  - How often did you share your PREVENT materials and program with your peers at your facility?
  - What has been the reaction of your peers to the PREVENT program at your facility?
- **Training** – What was your strategy for training your local staff on the PREVENT program and materials during sustainability phase?
    - Were there any strategies for sustaining a training program to address new staff and provider turnover?
- **Motivation to participate**: Are you motivated to sustain PREVENT? Why?
  - Has this changed over the course of the program – why?
  - How prepared do you feel to sustain PREVENT and to contribute to care of TIA patients (self-efficacy)?

Commented [PLS27]: Unrelated? Maybe ask above with questions about adoption?

Commented [PLS28]: Move above with questions specifically about sustainability (eg successes, challenges)

COMMUNITY OF PRACTICE

- Have you participated in any virtual/online professional community during the past few months of the sustainability phase of PREVENT? (If yes, which communities (PREVENT, others?)
  - What did you like about those?
  - What did you not like?
  - Ideally, what do you think are essential components to a thriving virtual community and learning collaborative?

One of the strengths of the PREVENT program was the opportunity to connect with others during the Virtual Collaborative Calls.

- Did you attend virtual collaborative calls after active implementation? If yes, why? If no, why not?

- Have you reached out to any of your colleagues across the PREVENT program during the sustainability phase?
  - Why or why not?
  - If Yes, what were some of the topics of those conversations?
    - Was there any shared learning that occurred during these interactions?

QUESTIONS FOR CHAMPIONS:

Looking back at the past 6 months,

- Have any other team members emerged as TIA champions? Who? Why do you think so?
- Who else has been active in the sustainability phase of PREVENT?
- Have you added new staff to the team?
- If so, what area do they serve (Nurse, Physician, ED, etc.)?

POLICY

- Currently, does your facility offer acute TIA care 24/7? During limited hours only – M-F 8-5? Has anything changed in the last 12 months?

- Does your facility have a written protocol or pathway for patients with suspected TIA?
    - If yes, please describe. [If only a stroke protocol, clarify that they do not have a specific TIA protocol].
    - If yes, where is it posted or kept?
    - Can you describe how it was developed during the yearlong implementation phase?

- How urgent do you view TIA care is now? [compared to prior PREVENT participation].

LEADERSHIP ENGAGEMENT-

- How has the local PREVENT team engaged with leadership [at level of service, facility, VISN, regional, national]?

- Does anyone from PREVENT team report to the Facility Director?
    - Are meeting notes submitted to the facility/VISN?

- How might implementation of a TIA program align (or conflict) with other organizational goals?
    - *PROMPT: For example, might it align with facility goals for risk factor management?*

- How important is the implementation of a TIA program to your organization's Executive Leadership? Why?

Commented [RNA29]: I wonder if these should be asked on page 1 of the interview guide. They are mostly descriptive, but will give participants a chance to answer factual questions first.

Commented [HB30]: Or to any standing committees: Acute Care, Critical Care, other?

Commented [HB31]: Additional prompts: SAIL, reducing readmissions, Chronic Disease/Health Promotion initiatives.

GENERAL QUESTIONS:

- Thinking about future PREVENT implementation, what advice would you give to personnel at a VAMC who want to implement PREVENT?
- Looking back, what would you have done differently if you knew then what you know now?
  - How do you think your team would have operated differently if you had another opportunity like this?
- What activity created the biggest impact on your PREVENT program during the sustainability phase?
- Have you been involved with any spread beyond ~~their~~ your facility? Ie – VISN meeting where they presented concerning the PREVENT program~~.~~?
  - Presentation about partner locations
  - Presentation to University setting (non-VA hospitals)
- Are you familiar with the ED-RAC or ED-PACT initiatives? [*Note: these are tools for ensuring patients leaving the ED with follow up needs receive appropriate, follow-up in PACT or specialty clinics*]
- What, if any, standardized processes do you have for addressing Veterans' care needs following ED visits when the patient has been discharged from the ED to home following a cerebrovascular incident?
  - Probes: Who does this (patient, ED provider, PCP, Neurologist, other person)? How is it done? When is it done (i.e., before leaving ED, after Veteran goes home)?
- What are some initiatives or future plans for your service and/or this facility in the next year related to TIA care?
- What other observations or comments do you have to share with us about current TIA care coordination at this facility?

Commented [PLS32]: Unclear – do we want to know if they've made presentations to these other groups?

Commented [PLS33]: Kristina Cordasco headed both these and they have similar intentions, so I think it'd be appropriate to ask about both.

Formatted: Font: Italic

Formatted: Font: Italic

Commented [HB34]: If interviewee is not familiar and is interested in learning more about these programs, materials are available on the Hub.
