# Supplementary material for: Acceptability of a complex team-based quality improvement intervention for transient ischemic attack: a mixed-methods study
Source: BMC Health Serv Res. 2021 May 12;21:453. doi: 10.1186/s12913-021-06318-2 (PMC8117601; doi:10.1186/s12913-021-06318-2)
Supplement: Supplementary file 4 — Additional File 4. [file 12913_2021_6318_MOESM4_ESM.docx]

Appendix A. Local PREVENT Quality Improvement Intervention Components designed to overcome systemic barriers to acute TIA care. (Damush, Miech, Rattray et al *Implementation Evaluation of a Complex Intervention to Improve Timeliness of Care for Veterans with Transient Ischemic Attack.* Journal of General Internal Medicine, 2021, Feb; 36(2) 322-332 doi 10.10071s 11606-020-06100.

**
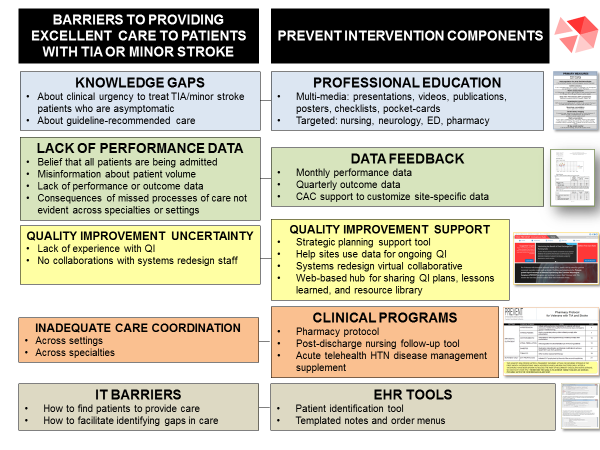
**
